# Latent profiles of Intelligence Quotient and Autism symptom severity in children with ASD

**DOI:** 10.1192/j.eurpsy.2025.1158

**Published:** 2025-08-26

**Authors:** Y. K. Lim, E. J. Chung, B. N. Kim

**Affiliations:** 1 Department of Child and Adolescent Psychiatry, Seoul National University Hospital, Seoul, Korea, Republic Of

## Abstract

**Introduction:**

Autism Spectrum Disorder (ASD) is a neurodevelopmental disorder characterized by difficulties in social communication and interaction, as well as restrictive and repetitive stereotyped behaviors, with considerable variation in cognitive and adaptive functioning. Accordingly, behavioral symptoms observed in daily life are likely to vary across individuals.

**Objectives:**

The present study aimed to explore the heterogeneity in intellectual abilities and behavioral issues among individuals with ASD using Latent Profile Analysis (LPA). This study was conducted between 2020 and 2021, with approval from the Institutional Review Board (IRB) of SNUH.

**Methods:**

A cross-sectional analysis was conducted on 66 children (ages 6-18) diagnosed with ASD. The following psychometric instruments were used: Autism Diagnostic Observation Schedule-2 (ADOS-2), Wechsler Intelligence Scale for Children-Fourth Edition (WISC-IV), Child Behavior Checklist 6-18 (CBCL 6-18), Social Responsiveness Scale (SRS).

**Results:**

As the 3-profile solution returned a significant BLRT value and model entropy was estimated to be 0.93, the LPA of the WISC-IV indices and ADOS-2 comparison scores revealed three profiles that were clinically meaningful and balanced in group sizes: Profile 1 (“Higher ASD score with intellectual disability”; ADOS = 6.25, VCI = 55.16, PRI = 52.34, WMI = 53.47, PSI = 50.31), Profile 2 (“Higher ASD score with borderline intelligence”; ADOS = 5.76, VCI = 78.59, PRI = 80.65, WMI = 80.82, PSI = 67.12), and Profile 3 (“Lower ASD score with above-average intelligence”; ADOS = 4.41, VCI = 105.53, PRI = 108.41, WMI = 106.35, PSI = 89.41). On the SRS subscales, Profile 3 showed significantly lower scores in Social Cognition, Social Communication, and Social Motivation compared to Profile 1 (*F*(2, 63) = 10.45, *p* <.001; *F*(2, 63) = 5.24, *p* < .01; *F*(2, 63) = 8.75, *p* < .001). Additionally, on the CBCL syndrome subscales, Profile 3 showed significantly lower problem behaviors in Withdrawal/Depression, Social Immaturity, and Attention Problems (*F*(2, 63) = 4.57, *p* <.05; *F*(2, 63) = 5.07, *p* < .01; *F*(2, 63) = 4.19, *p* < .01).

**Conclusions:**

In the present study, Latent Profile Analysis (LPA) using IQ and ADOS scores identified three distinct profiles within children with ASD. The findings suggest that while high-functioning ASD has traditionally been defined by an IQ threshold of 70–75, further distinction between borderline and above-average intelligence ASD groups may be warranted. Furthermore, targeted interventions addressing negative emotions, such as depression, may be indicated for the ASD group with intellectual disability.

**Disclosure of Interest:**

Y. K. Lim Financial Support for Research from:, Financial Support for Research from: This research was supported by the National Research Foundation (NRF) funded by the Korean Government (MSIT) (RS-2024-00397737), and co-funded by the National IT Industry Promotion Agency(NIPA), an agency under the MSIT and with the support of the Daegu Digital Innovation Promotion Agency (DIP), the organization under the Daegu Metropolitan Government., E. Chung: None Declared, B. N. Kim: None Declared

